# Relation between insulin growth factor 1 and survival after SARS-CoV-2(COVID 19) infection in elderly kidney transplant recipients

**DOI:** 10.1080/0886022X.2021.1886115

**Published:** 2021-02-24

**Authors:** Amin Roshdy Soliman, Khaled Marzouk Sadek

**Affiliations:** Nephrology Division, Internal Medicine Department, Kasrainy School of Medicine, Cairo University

Dear Editor,

We report our work on the association of two important biomarkers (serum Insulin-like growth factor −1(IGF-1) and human growth hormone (HGH) with adverse outcomes in kidney transplant recipients with confirmed SARS-CoV-2 infections.

Insulin-like growth factor −1 is a biomarker in patients with hyperoxia induced lung injury. IGF 1 levels are elevated in lung biopsy specimens in ARDS compared to those in normal persons [1]. The serum levels of IGF-1 and Insulin Growth Factor Binding Protein −3 (IGFBP-3) were found to be elevated in patients who developed early Adult Respiratory Distress Syndrome (ARDS), when there is death and damage of epithelium. However, Their level was low in late ARDS.

Growth hormone releasing peptide-2 (GHRP-2), a ghrelin agonist, has been shown to exert beneficial effects on various inflammatory diseases. It was explored whether GHRP-2 possesses anti-inflammatory properties in the pathogenesis of lipopolysaccharide (LPS)-induced acute lung injury (ALI) in experimental rats [2]. The complexity of lungs, muscles, and systemic inflammatory responses of acute lung injury (ALI) that may be time-dependent, organ-dependent, and individualized [3].

We have previously published our work on the association of serum Insulin-like growth factor −1 and growth hormone(GH) levels in elderly kidney transplant recipients with cardiac dysfunction) [4].

We extended our work to explore the association of serum growth hormone(GH) and Insulin-like growth factor −1 (IGF1) levels on adverse outcome and mortality complicating (SARS-CoV-2) infection in elderly renal transplant recipients

Our study was conducted on 53 elderly kidney transplant recipients. Sixteen patients had SARS-CoV-2, confirmed by PCR . Patients with confirmed SARS-CoV-2 were divided into two groups:

- Group 1 (patients with no cardiac dysfunction) that included nine patients.
- Group 2 (patients with cardiac dysfunction) that included seven patients.

Quick sepsis-related organ failure assessment score (Q-SOFA) score was calculated for patients with positive (SARS-CoV-2) who had been admitted to the Intensive Care Unit (ICU). Q-SOFA score is an important score for evaluation of clinical course of ICU patients. A high Q-SOFA score is associated with worse adverse clinical outcome and higher incidence of mortality.

The levels of both IGF1 and HGH were measured at 2 different time points

1. Baseline levels (Before developing infection with (SARS-CoV-2).
2. After confirmation of SARS-CoV-2 infection.

During the follow up of the fifty-three elderly renal transplant recipients; sixteen recipients had confirmed SARS-CoV-2 infection. They were 12 males and 4 females with mean age 75.71 ± 2.43 in Group 1 (patients without cardiac dysfunction) and mean age 76.56 ± 2.3 in Group 2 (patients with cardiac dysfunction).

Baseline human growth hormone (HGH) levels were higher in group 2 compared to group 1 (P value 0.001) (Table 1). Furthermore, after SARS-CoV-2 infection, HGH was higher in Group 2 than in Group 1(P value 0.00 (Table 1). Before developing infection with (SARS-CoV-2), Insulin Growth Factor 1 (IGF1) was higher in Group 1 than in Group 2 (P value 0.001) (Table 1).

**Table 1.** Laboratory data of elderly renal transplant recipients with and without cardiac dysfunction before and after Covid 19 infection.

	Group one (Patients without cardiac dysfunction)	Group two (Patients with cardiac dysfunction)	*p* Value
Human growth hormone before(SARS-CoV-2)			
Mean ± SD	1.27 ± 0.18	4.35 ± 1.39	<0.001
Median(IQR)	1.3(1.1:1)	4.9(3.8:5)	
Human growth hormone after (SARS-CoV-2)			
Mean ± SD	1.21 ± 0.16	3.3 ± 0.99	<0.001
Median(IQR)	1.2(1.1:1)	2.8(2.5:4)	
Insulin Growth Factor 1 (IGF1) before (SARS-CoV-2)			
Mean ± SD	190.57 ± 19.26	67.44 ± 21.24	<0.001
Median(IQR)	196(179:205)	60(52:85)	
IGF1 after (SARS-CoV-2)			
Mean ± SD	209.71 ± 48.4	129.78 ± 39.43	0.002
Median(IQR)	195(190:225)	123(98:150)	

Spearman's rho	Q sofa score	
	*r*	*p* Value
Group without cardiac dysfunction		
IGF1 before Covid19	0.408	0.363
IGF1 after Covid19	0.618	0.139
Group with cardiac dysfunction		
IGF1 before Covid19	−0.149	0.702
IGF1 after Covid19	0.894	0.001

After developing (SARS-CoV-2), IGF1 levels were higher in Group 1 than in Group 2 (P value 0.001) (Table 1). IGF1 was lower in those patients recovering from (SARS-CoV-2) infection in Group 1 (patients without cardiac dysfunction), (Table 2). IGF1 was lower in those patients recovering from (SARS-CoV-2) infection, especially in Group 2 with cardiac dysfunction (Table 2). The high level of IGF1 was associated with high Q- SOFA score in both groups of patients.

**Table 2.** Relation between recovery and HGH and IGF 1 in elderly transplanted recipients with and without cardiac dysfunction.

	Recovery		
	No	Yes	*p* Value
Group without cardiac dysfunction			
IGF1 after (SARS-CoV-2)			
Mean ± SD	310±.	163 ± 21.54	0.02
Median(IQR)	310(310:310)	162.5(160:170)	
Group with cardiac dysfunction			
IGF1 after (SARS-CoV-2)			
Mean ± SD	166.25 ± 22.49	100.6 ± 18.31	0.016
Median(IQR)	164(147.5:185)	98(88:115)	
Group without cardiac dysfunction			
Human growth hormone after Covid19			
Mean ± SD	1±.	1.25 ± 0.14	0.286
Median(IQR)	1(1:1)	1.2(1.2:1.3)	
Group with cardiac dysfunction			
Human growth hormone after Covid19			
Mean ± SD	2.88 ± 0.99	3.64 ± 0.94	0.19
Median(IQR)	2.5(2.2:3.6)	3.9(2.8:4.4)	

	Ventilation		*p* Value
	No	Yes	
Group without cardiac dysfunction			
IGF1 after (SARS-CoV-2)			
Mean ± SD	186.6 ± 16.52	267.5 ± 60.1	0.095
Median(IQR)	190(190:195)	267.5(225:310)	
Group with cardiac dysfunction			
IGF1 after (SARS-CoV-2)			
Mean ± SD	97 ± 18.99	156 ± 30.07	0.032
Median(IQR)	93(83.5:111)	150(145:178)	

HGH was higher in Group 2 than in Group 1 before and after infection (P value 0.001) (Table 1). HGH was higher in patients that recovered from (SARS-CoV-2) than the non-survivors in Group 1 (without cardiac dysfunction) after getting infection (P value 0.286) (Table 2). HGH was higher in patients recovering from (SARS-CoV-2) compared to non-survivors in Group 2 (patients with cardiac dysfunction) after getting infection (P value 0.19) (Table 2).

We found that Insulin Growth Factor 1 (IGF 1) was lower among the survivors after (SARS-CoV-2) infection in the elderly renal transplanted patients with or without cardiac dysfunction. In previous studies, it was found that levels of IGF-1 and IGFBP-3 were elevated in at-risk patients and those with early ARDS, indicating epithelial damage. However, they decreased in late ARDS [5]. ARDS is strongly associated with IGF-1 and IGFBP-3 levels in critically ill patients [6].

In our study we found that, before developing infection with (SARS-CoV-2), human growth hormone (HGH) was higher in Group 2 than in Group 1 (P value 0.001) (Table 1). Also, after getting infection with (SARS-CoV-2), HGH was higher in Group 2 than in Group 1 (P value 0.001) (Table 1). Before developing infection with (SARS-CoV-2), Insulin Growth Factor 1 (IGF1) was higher in Group 1 than in Group 2 (P value 0.001) (Table 1). Moreover, after getting infection with (SARS-CoV-2), IGF1 was higher in Group 1 than in Group 2 (P value 0.001) (Table 1).

It was shown in a previous prospective case-control study that baseline plasma levels of IGF-1 and IGFBP-3 had significant lower level in ARDS cases than in controls [6]. Among ARDS cases, Levels of IGF-1 and IGFBP-3 were lower in patients who did not survive than in the survivors, and both groups were negatively associated with risk of 60-day mortality [7]. Dynamic assay revealed that plasma IGF-1 level is higher on day 1, but lower on day 3 in ARDS cases associated with severe pneumonia. The level of Insulin Growth Factor 1 Receptor (IGF-1R) in plasma is higher on days 1, 3, and 7 in ARDS cases associated with severe pneumonia than level in the normal controls [8].

IGF1 was lower in those patients recovering from (SARS-CoV-2) infection in Group 1 (patients without cardiac dysfunction), especially after getting infection (P value 0.02) (Table 2). IGF1 was lower in those patients recovering from (SARS-CoV-2) infection, especially in Group 2 with cardiac dysfunction, especially after developing infection (P value 0.016) (Table 2). IGF1 was lower in those patients recovering from (SARS-CoV-2) infection, especially in Group 2 with cardiac dysfunction, especially after infection (P value 0.016) (Table 2). The high level of IGF 1 was associated with high Q Sofa score in both groups of patients.

It was reported that, throughout the course of ARDS, There were dynamic changes in the levels of Insulin Growth Factor Binding Protein −4 (IGFBP-4) and IGFBP-2 providing them to be a possible biomarker [7].

HGH was higher in Group 2 than in Group 1 before and after developing infection (P value 0.001) (Table 1). HGH was higher in those patients recovering from (SARS-CoV-2) than in the non-survivors in Group 1 (without cardiac dysfunction) after getting infection (P value 0.286) (Table 2). HGH was higher in those patients recovering from (SARS-CoV-2) than in the non-survivors in Group 2 (patients with cardiac dysfunction) after getting infection (P value 0.19) (Table 2). Higher HGH in patients who did not survive SARS-CoV-2 may support previously published findings of HGH resistance in ARDS [3].

Insulin Growth Factor 1 (IGF 1) was lower among survivors after (SARS-CoV-2) infection in the elderly renal transplanted patients while level of Human Growth hormone (HGH) was higher among the survivors after (SARS-CoV-2) infection in the elderly renal transplanted patients and may be protective against ADRS and late pneumonia after SARS-CoV-2 infection for further study for evaluation.
